# Draft Genome Sequence of *Geobacillus subterraneus* Strain K, a Hydrocarbon-Oxidizing Thermophilic Bacterium Isolated from a Petroleum Reservoir in Kazakhstan

**DOI:** 10.1128/genomeA.00782-16

**Published:** 2016-08-04

**Authors:** Andrey B. Poltaraus, Diyana S. Sokolova, Denis S. Grouzdev, Timophey M. Ivanov, Sophia G. Malakho, Alena V. Korshunova, Tatiyana P. Tourova, Tamara N. Nazina

**Affiliations:** a Engelhardt Institute of Molecular Biology, Russian Academy of Sciences, Moscow, Russian Federation; b Research Center of Biotechnology of the Russian Academy of Sciences, Moscow, Russian Federation; c Federal State Budgetary Institution, “Federal Science Center for Physical Culture and Sport,” Moscow, Russian Federation

## Abstract

The draft genome sequence of *Geobacillus subterraneus* strain K, a thermophilic aerobic oil-oxidizing bacterium isolated from production water of the Uzen high-temperature oil field in Kazakhstan, is presented here. The genome is annotated for elucidation of the genomic and phenotypic diversity of thermophilic alkane-oxidizing bacteria.

## GENOME ANNOUNCEMENT

*Geobacillus subterraneus* strain K (=VKM B-2225) was isolated from a formation water sample of the Uzen high-temperature oil field (Kazakhstan) (44°44′33.3″ N, 52°78′22.2″ E) and was described as a reference strain of the species (1). *G. subterraneus* strain K is an aerobic endospore-forming bacterium able to grow at temperatures ranging from 48 to 70°C with an optimum at 60°C and utilizing fatty acids, sugars, amino acids, aromatic compounds, and *n*-alkanes of crude oil. In the genome of strain K growing on C_16_ and C_22_ *n*-alkanes, a range of *alk*B gene homologs was revealed by PCR amplification (2). The *alk*B and *ladAB* genes coding the monooxygenases associated with *n*-alkane oxidation have been detected in several thermophilic bacilli (3–5).

Genomic DNA was isolated from the biomass using the DNeasy Blood & Tissue Kit (Qiagen, Germany) according to the manufacturer’s instructions. The TruSeq DNA Sample Preparation Kit (Illumina, United States) was used to create the libraries for genome sequencing. Genomic DNA was sequenced using the MiSeq Reagent Kit version 2 (Illumina). The shotgun library was constructed with a 500 bp-span paired-end library.

Approximately 11,888,884 paired-end reads were generated providing 250-fold genome coverage. The resulting reads were assembled with SPAdes version 3.1.0 (6). The remaining 158 contigs were submitted to the ProDeGe website (7) for automatic decontamination. The assembled sequence was submitted to the National Center for Biotechnology Information (NCBI) Prokaryotic Genome Annotation Pipeline (PGAP) (8) for annotation.

The draft genome sequence of *G. subterraneus* strain K revealed a genome size of 3,309,142 bp, with an average G+C content of 51.43%, slightly exceeding the 49.7% value determined earlier (1). The genome contained 3,275 genes, of which 3,058 were coding DNA sequences, 18 coded rRNAs (5S, 16S, and 23S), 70 coded tRNAs, and five belonged to noncoding RNAs (ncRNAs). There were no complete genes coding 16S rRNA, but the *gyrB* and *parE* gene sequences revealed in the genome had 99.9% and 99.6% similarity, respectively, with those determined earlier for the strain (9). The results of phenotypic studies characterizing the strain as an aerobic spore-forming bacterium utilizing a wide range of organic substrates were confirmed by the detection of respective genes. Despite utilization of a range of *n*-alkanes by strain K, neither *alk*B nor *ladA* genes were found in the draft genome. A range of protein-coding sequences involved in the *β*-oxidation pathway was detected in the genome of the strain K. Among them were alcohol dehydrogenases, aldehyde dehydrogenase, long-chain fatty acid-CoA ligases, acyl-CoA dehydrogenase, enoyl-CoA hydrogenase, hydroxyacyl-CoA dehydrogenase, and thiolase. Further studies of the genes of alkane biodegradation by aerobic thermophilic bacteria are necessary.

### Accession number(s).

This whole-genome shotgun project has been deposited at DDBJ/ENA/GenBank under the accession no. LWHE00000000. The version described in this paper is version LWHE01000000.
